# Combined isochoric processes of freezing and supercooling

**DOI:** 10.1038/s41538-025-00542-4

**Published:** 2025-08-24

**Authors:** Cristina Bilbao-Sainz, Boris Rubinsky

**Affiliations:** 1 U.S. Department of Agriculture, Western Regional Research Center, 800 Buchanan St., Albany, CA 94710 USA; 2 Department of Mechanical Engineering, University of California, 6141 Etcheverry Hall, Berkeley, CA 94720 USA

**Keywords:** Engineering, Physics

## Abstract

This study presents a thermodynamic analysis and design strategy for a multiphase isochoric system that enables supercooled preservation of matter at lower temperatures without increasing the probability of ice nucleation. In isochoric supercooling, ice nucleation events follow a Poisson distribution and depend on the temperature differential between the equilibrium phase transition temperature and the preservation temperature. The proposed technology employs a multiphase isochoric system in which matter, suspended in an isotonic solution, is enclosed within a compartment bounded by a membrane that permits heat and pressure exchange but prevents mass transfer. The remaining chamber volume is filled with water. Preservation begins by cooling the system until the water phase fully freezes, inducing a pressure rise. The biological compartment is then supercooled relative to the new equilibrium pressure and temperature established by the frozen water. This capability enables the design of elevated-pressure conditions that reduce microbial contamination without compromising quality.

## Introduction

This study explores the combined processes of freezing and supercooling in an isochoric (constant-volume) system. Research on freezing in closed volumes is not new and has been investigated across various fields. It is well known that a sealed bottle completely filled with water will explode upon freezing. This phenomenon occurs because ice Ih, which forms within the temperature range of 0 °C to ~–21 °C, has a lower density than liquid water. Consequently, it expands during freezing, generating pressures that can reach up to 220 MPa. This effect has been extensively studied in engineering for over a century.

Similar phenomena have been observed in various contexts, including the freezing of water pipes in winter^1^, soil freezing^2^, and the freezing of concrete^3,4^. Hayakawa et al. proposed that the pressure generated by freezing water in a sealed vessel could be utilized for bacterial destruction^5^. Their experiments demonstrated that microorganisms in sealed vessels frozen to −20 °C (theoretically corresponding to 200 MPa) for 24 h were completely eradicated, whereas microorganisms in open vessels subjected to the same freezing conditions survived^5^.

The concept of using freezing in sealed capillaries to generate high pressures for electron microscopy was introduced by Hans Moor and Ueli Riehle at the 1968 European Conference on Electron Microscopy in Rome^6,7^. More recent studies have explored freezing in sealed capillaries as an alternative to high-pressure freezing, with findings documented by in ref. ^8^ and ref. ^9^.

The novelty of our research stems from rigorous thermodynamic analyses that have enhanced the understanding of processes occurring in an isochoric system during freezing within the temperature range from 0 °C to the triple point of water, ice Ih, and ice III^10–16^. These thermodynamic studies of isochoric (constant-volume) systems at subfreezing temperatures have led to the development of several novel technologies for preserving biological materials, food, and biomedical samples in an unfrozen state at subfreezing temperatures (e.g., refs. ^10,12–30^) The principles derived from these thermodynamic analyses have led to various applications in the preservation of biological matter, drawing directly from these findings (e.g., refs. ^18,19,31–56^) Several reviews of isochoric processing technology can be found in refs. ^57–60^.

**Fig. 1:**
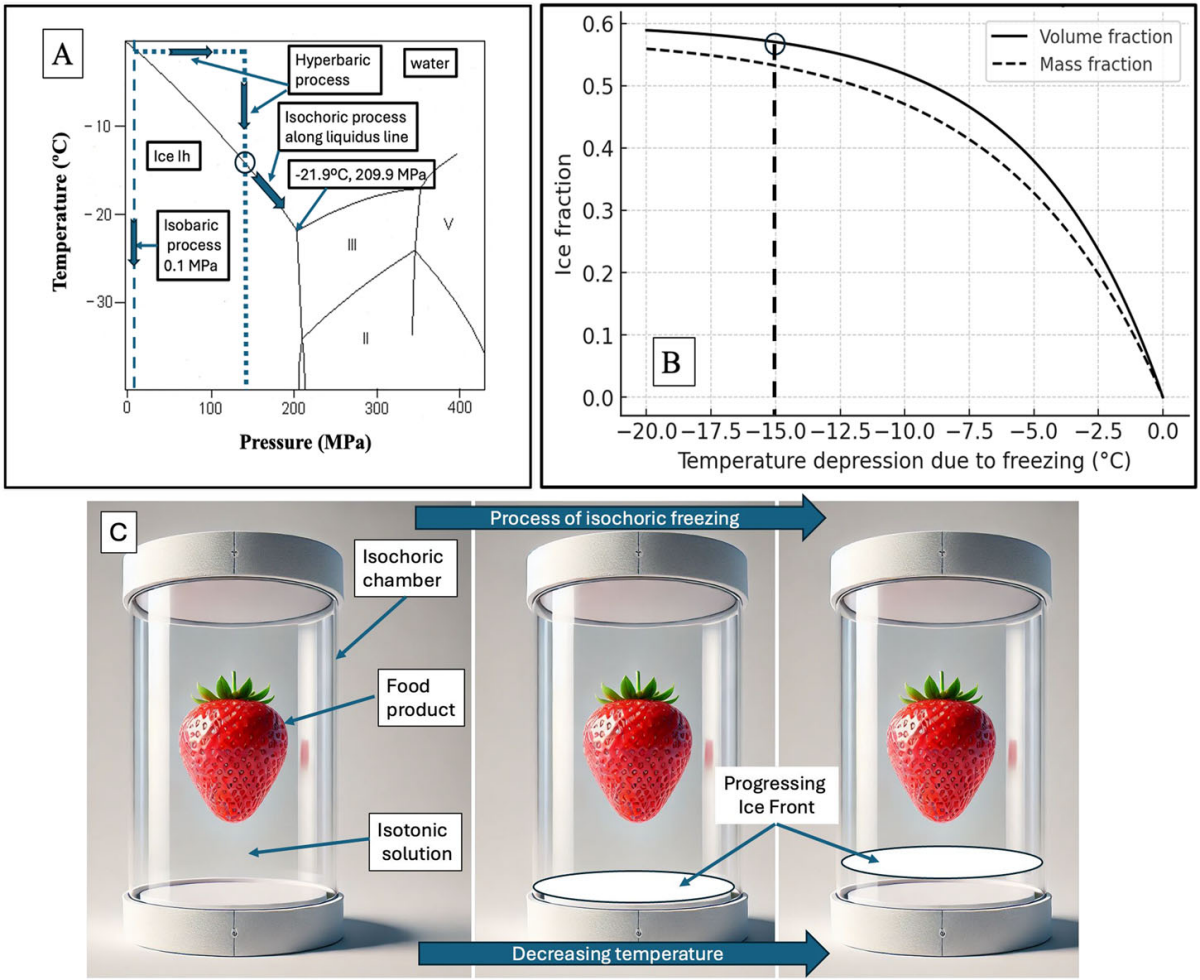
The principles of the isochoric freezing process. The priciples of the isochoric freezing process are illustrated in **A** and **B**, while **C** demonstrates the preservation of biological matter in the unfrozen portion of a frozen isochoric system. Specifically, **C** depicts the freezing process initiated by an ice nucleation site positioned at the base of the chamber.

The thermodynamic analysis of freezing in an isochoric system has shown that as the temperature decreases below the freezing point, the freezing process follows the liquidus line, which separates water and ice Ih. Consequently, the system consists of two phases in thermodynamic equilibrium: ice Ih and liquid water. As the temperature continues to drop toward the triple point (−21.9 °C and 209.9 MPa), both the system pressure and the fraction of ice increase. A substantial portion of the volume remains unfrozen throughout the freezing process, even at the triple point. This observation led us to a key hypothesis: when biological matter is sequestered within the unfrozen portion of the isochoric system, it can be maintained at subfreezing temperatures in an unfrozen state, thereby avoiding the detrimental effects of ice formation^10^. Figure 1 illustrates the key findings from the analysis in ref. ^10^, which led to the development of the subzero isochoric (constant-volume) processing concept.

Figure 1A presents a segment of the water phase diagram, focusing on the liquidus line between water and ice Ih. It depicts the isochoric freezing process along the liquidus line and demonstrates how system pressure increases as temperature decreases. In an isochoric system undergoing freezing, temperature and pressure are interdependent thermodynamic properties. Along the liquidus line, from 0 °C to the triple point, liquid water and ice Ih remain in continuous thermodynamic equilibrium, with a substantial portion of water remaining unfrozen throughout the process. Figure 1B was replotted from several of our own calculations in [10] and depicts the volume fraction and mass fraction of ice in an isochoric system of pure water along the liquidus line as a function of temperature, extending down to the triple point. This figure supplements the mass fraction plot in [10] by adding the volume fraction to highlight the distinction between mass fraction and volume fraction, which arises due to the density difference between ice Ih and water.

Notably, even near the triple point, approximately 40% of the water remains unfrozen. This observation forms the basis of our proposal that biological matter can be preserved within this unfrozen fraction during the freezing process in an isochoric system—a concept that had not been previously recognized.

The principle of preservation within the unfrozen portion of a frozen isochoric system is illustrated in Fig. 1C^10,13,17^. This figure depicts biological matter in an unfrozen state, sequestered within the unfrozen portion of a partially frozen isochoric system. The direction of freezing in the system is determined by the location of the nucleation site and the thermodynamic preference of water molecules to attach to existing ice crystal facets. Once nucleation occurs, ice propagation proceeds outward from the nucleation site into the surrounding supercooled liquid. In the configuration illustrated in Fig. 1C, we assume that nucleation initiates at the bottom of the chamber. Consequently, the freezing front advances upward from this location through the bulk of the fluid. In many practical implementations, the nucleation site is deliberately positioned at a location well separated from the biological specimen. A nucleation site can be introduced by placing a solid material with high surface energy and irregular boundaries at the bottom of the chamber. While metals are commonly used due to their thermal conductivity and structural rigidity, other materials, such as ceramics or roughened glass, can also serve effectively. For instance, a piece of mechanically abraded stainless steel is frequently employed. The rugged features and microcavities on these surfaces promote heterogeneous ice nucleation by providing energetically favorable sites for the formation of a critical ice embryo. According to classical nucleation theory, such surface features reduce the Gibbs free energy barrier required for nucleation by locally stabilizing molecular clusters and enhancing the solid–liquid interfacial interaction^61,62^ .

It is important to clarify that referring to water in an ice-water mixture along the liquidus line as “supercooled” is thermodynamically incorrect. At this point, the water is at its freezing temperature, corresponding to the specific pressure and volume, and exists in stable thermodynamic equilibrium. When biological matter is preserved within the unfrozen portion of an isochoric system along the liquidus line, it remains in an unfrozen state that is also in stable thermodynamic equilibrium. The system only becomes supercooled when the biological matter is at a temperature below the liquidus line for a given pressure, indicating a metastable state rather than equilibrium. Figure 1A, B also highlight the key differences between freezing processes in isochoric and hyperbaric systems. In a hyperbaric system, freezing occurs in two distinct steps: first, the pressure is mechanically increased, and second, the system is cooled. When the constant-pressure line intersects the liquidus line (marked with a circle), the entire volume of water freezes instantaneously. In contrast, in an isochoric system, freezing progresses along the liquidus line as the system cools, with the pressure increasing naturally without mechanical intervention. As the temperature continues to decrease, freezing follows the liquidus line. At the same intersection point (marked with a circle in Fig. 1B), where complete freezing occurs in a hyperbaric system, 42% of the water in an isochoric system remains unfrozen. This retained unfrozen fraction enables the preservation of biological matter within the unfrozen portion, forming the basis of isochoric processing by freezing.

The thermodynamic processes in isochoric systems at subfreezing temperatures offer a wealth of opportunities for fundamental research and applications—many of which remain unexplored. Our experimental and thermodynamic studies on isochoric systems at subfreezing temperatures have revealed another valuable feature with significant implications for the preservation of biological materials at subfreezing temperatures without ice formation. Specifically, we found that maintaining an isochoric, constant-volume thermodynamic state significantly enhances the stability of supercooled systems^11,15,22,63^. To support this research, we developed a nucleation detection system^64^ and a statistical technique to predict the probability of ice nucleation in isochoric systems based on the degree of supercooling and storage duration^26^.

Several mechanisms contribute to the stabilization of supercooling in isochoric systems. One key factor is their unique thermodynamic behavior, where ice nucleation is governed by Helmholtz equilibrium and Le Chatelier’s principle^11,13–15^. Another critical stabilizing mechanism is the suppression of cavitation-induced nucleation^27^.

**Fig. 2:**
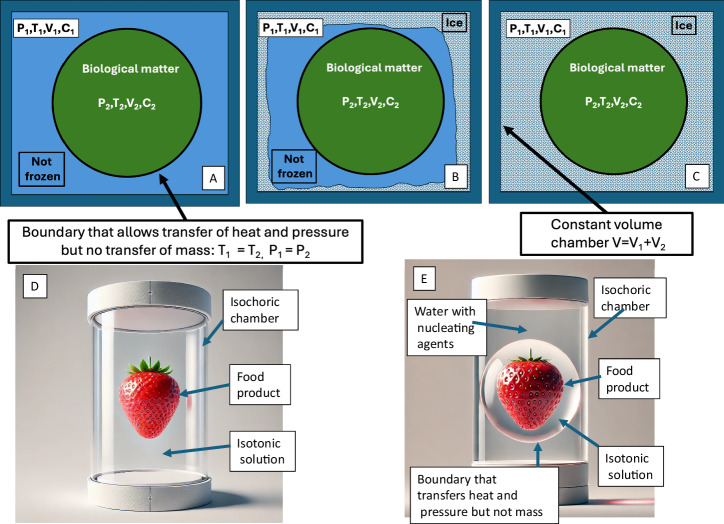
A schematic of the process of supercooling in this study. **A** Schematic of an isochoric system in which the biological matter is encased in a boundary that transmits pressure and heat but is impermeable to mass surrounded by water that completely fills the isochoric chamber. **B** Onset of the process of freezing from the boundaries of the isochoric chamber. **C** End of the freezing process in which the water surrounding the biological matter has frozen completely, while the biological matter is unfrozen. **D** Illustration of a food product in an isochoric chamber, in a conventional isochoric freezing process. The food product is in direct contact with the fluid that fills the chamber and mass transfer between the surrounding fluid and the food product is possible. **E** Configuration analyzed in this paper in which the food product is surrounded by a boundary that transfer pressure and heat but is impermeable to mass transfer.

Isochoric supercooling has already demonstrated potential in diverse applications. In medicine, it has been used to preserve livers for transplantation^51,52^, heart tissue chips for drug development^65^, and organoids for delivering live biological matter to the space station^66^. Additionally, it has proven effective in preserving food products, e.g., ref. ^36^. This study builds on the isochoric supercooling work of refs. ^25,26,64,67^ and explores the combined effects of isochoric freezing^10,17,19^ and isochoric supercooling as a novel technology for the preservation of biological matter. The proposed approach employs a multiphase isochoric system and a combination of isochoric freezing and isochoric supercooling to lower the storage temperature of supercooled biological matter without increasing the probability of ice nucleation. Figure 2 and its accompanying description illustrate the underlying freezing process and the configuration of the multiphase isochoric system.

Figure 2A depicts the system setup. The biological matter is surrounded by a solution in osmotic equilibrium with the matter, jointly enclosed in a separate compartment, within a flexible, impermeable boundary. This boundary permits the transfer of heat and pressure but prevents mass exchange, thereby preserving the composition of the internal solution in the separate compartment throughout the isochoric preservation process. Importantly, the boundary is constructed from materials that inhibit ice nucleation. Unlike nucleation-promoting surfaces, nucleation-inhibiting materials are typically hydrophobic and lack surface features or chemical functionalities that facilitate the ordering of water molecules into an ice-like lattice. Hydrophobic polymers such as polytetrafluoroethylene (PTFE) and silicone elastomers are commonly used to reduce the likelihood of heterogeneous nucleation. The nucleation suppression is attributed to unfavorable wetting conditions and limited hydrogen bonding at the water–surface interface, which raise the Gibbs free energy barrier for critical ice embryo formation. Incorporating such hydrophobic materials in the compartment boundary enhances nucleation resistance and helps maintain metastable supercooled conditions throughout the proposed isochoric preservation process.

The compartment containing the biological matter is surrounded by an external solution that completely fills the rest of the isochoric chamber and that nucleates and freezes before the internal solution in the separate compartment begins to freeze. To achieve this, the external solution must have a higher freezing point than the isotonic solution inside the boundary. Although various formulations could be used for the external surrounding solution, pure water is preferred due to its simplicity and ease of implementation, particularly when compared to the more complex requirements of isochoric freezing in solution^10^. For this reason, the external chamber is preferably completely filled with pure water, which fully occupies the chamber volume. In the process analyzed here, it is essential that the external solution freezes immediately upon cooling, without undergoing supercooling. One strategy to ensure this behavior is to fabricate the chamber walls from materials that promote ice nucleation or to incorporate a designated ice-nucleation site, as described earlier in the introduction. An additional technique is to add ice-nucleating agents directly to the external water. Common nucleating agents used in this system include commercially available ice-nucleating proteins such as *InaZ* from *Pseudomonas syringae*. A widely used commercial formulation is Snomax® (York Snow, Inc.), which contains freeze-dried preparations of *P. syringae* and has been shown to initiate ice formation at relatively high subzero temperatures^68^.

Figure 2B illustrates the initial stage of the freezing process as the system temperature decreases. Ice first forms in the water external to the compartment boundary, initiated by the presence of ice-nucleating proteins and heterogeneous nucleation at the chamber walls. As cooling continues, freezing propagates through the water, and the pressure within the system rises due to the isochoric constraint. Because the boundary enclosing the biological material permits the transfer of heat and pressure, but not mass, the temperature and pressure within the entire isochoric chamber remain uniform. This rise in pressure further depresses the thermodynamic equilibrium freezing point of the solution and of the biological matter inside the compartment, thereby eliminating the likelihood of ice formation around the biological matter.

Figure 2C depicts the system when the entire external aqueous phase, pure water surrounding the compartment containing the biological matter, has frozen. The system is specifically engineered so that, at the moment the ice front reaches the compartment boundary, the internal pressure reaches the maximum level predetermined for the intended preservation process. At that stage, the biological matter is in an unfrozen thermodynamic state, for the particular combination of pressure and temperature in the system, and the solute composition. The relevant mathematical modeling of these thermodynamic conditions will be presented in the Results and Discussion and Methods sections. As the temperature is further reduced, the biological matter and the isotonic solution within the compartment eventually enter a supercooled state, relative to the equilibrium conditions established when the surrounding water became completely frozen and the resulting maximal pressure was reached. Because the boundary is impermeable to mass, ice propagation halts at its surface, even as the external temperature continues to drop. This physical barrier effectively prevents ice nucleation or growth within the internal compartment. As cooling continues, the contents within the boundary remain supercooled without freezing, until random nucleation ensues. The mathematical analysis in the Results and Discussion and Methods sections will demonstrate that, for the same probability of ice nucleation, the storage temperature achievable in a multiphase isochoric system can be significantly lower than that in a single-phase isochoric system. The design in this study enables precise thermodynamic control over the freezing process and allows biological matter to be preserved at subfreezing temperatures at a prescribed pressure, temperature and composition in a metastable, ice-free state. Such control is particularly advantageous for the preservation of sensitive biological samples, such as cells, tissues, or organs, as well as high-value food products, where ice formation would otherwise compromise structure, function, or quality.

There are several important differences between the system analyzed in this study and those investigated in our previous work^25,26,64,67^. In contrast to the idealized systems composed of pure water examined in ref. ^67^, the present study addresses a more practical and realistic application involving biological matter immersed in an isotonic solution. Moreover, unlike the scenarios described in refs. ^10,17,69^, where the freezing process alters the composition of the solution surrounding the biological matter, due to the exclusion of solutes from the ice phase, the system analyzed here preserves a constant chemical environment throughout the preservation process. This is achieved by enclosing the biological matter and its surrounding isotonic solution within a boundary that allows the transfer of heat and pressure but prevents mass exchange. As a result, the composition of the solution surrounding the biological material remains unchanged during the freezing process.

Importantly, the aqueous phase external to the boundary does not need to be isotonic to the biological matter and may preferentially consist of pure water. The chemical stability of the internal compartment significantly simplifies the analysis of the system compared to earlier isochoric configurations, where dynamic changes in solute concentration during freezing complicated the thermodynamic modeling^10^. This compositional stability is a key advantage of the proposed approach.

By adjusting the volume of pure water in the external chamber and freezing the system to the equilibrium freezing point corresponding to that volume, the internal pressure of the isochoric system can be regulated independently of the composition within the biological compartment. This decoupling of pressure from the isochoric supercooling temperature allows for separate control of pressure and the degree of supercooling. As a result, the system can be designed to achieve elevated pressures that effectively reduce microbial contamination in food without compromising food quality.

## Results and discussion

The isochoric freezing and isochoric supercooling process proceeds as follows: As the isochoric chamber is cooled below the freezing temperature of pure water, ice begins to form in the external aqueous phase, which surrounds the compartment containing the biological matter. This initial ice formation leads to a rise in system pressure due to the isochoric constraint. Once the advancing ice front reaches the boundary of the internal compartment, further freezing is halted, and the pressure increase ceases. At this point, the total volume of pure water in the system dictates the equilibrium pressure at temperatures below the threshold where the external water becomes fully frozen.

The equilibrium freezing temperature of the solution inside the compartment is determined by both its composition and the prevailing system pressure. When the temperature of the entire isochoric chamber is subsequently lowered below this pressure-dependent equilibrium temperature, the internal solution enters a supercooled state. The design illustrated in Fig. 2 enables precise thermodynamic control of this process, allowing the biological matter to be preserved at subfreezing temperatures in a metastable, ice-free condition while maintaining defined pressure conditions. By carefully selecting the solute composition and pressure within the compartment, it is possible to tailor the equilibrium freezing temperature of the system. Notably, pressure can be finely regulated by adjusting the volume of pure water in the external phase, providing an independent control variable for system design and optimization.

In this section, we present illustrative examples demonstrating how to design a supercooling system based on the approach outlined in the Introduction.

**Fig. 3:**
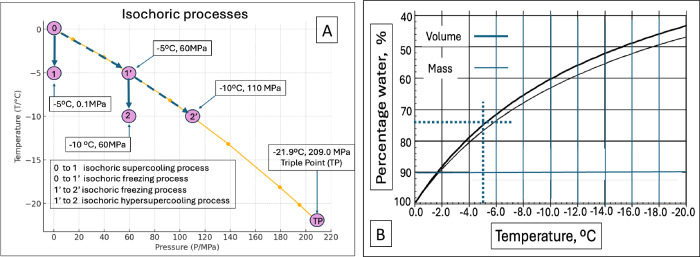
The thermodynamic processes of supercooling, isochoric freezing, the combination of isochoric freezing and supercooling and hyperbaric freezing. **A** Temperature pressure phase diagram for water at the liquidus line which separates between the domain of pure water, above the line and ice Ih below the line. The line represents the thermodynamic state at which water and ice Ih are in thermodynamic equilibrium. The panel illustrates the thermodynamic path for different isochoric processes, isochoric supercooling, isochoric freezing and isochoric hypersupercooling (introduced in this study). **B** Calculated percentage volume and mass of unfrozen water in a freezing isochoric system, as a function of the temperature of the system. (Modified from^17^ with permission).

Figure 3A illustrates the thermodynamic processes central to this study: the isochoric supercooling process (ISP), the isochoric freezing process (IFP), and the isochoric freezing supercooling process (IFSP), along with their corresponding thermodynamic states. This figure was developed specifically for pure water and provides a comprehensive visualization of how these processes interact within an isochoric system. It serves as a critical reference for understanding the relationships between temperature, pressure, and phase transitions in isochoric preservation.

The isochoric supercooling process (ISP) begins on the liquidus line at atmospheric pressure (0.1 MPa) and progresses along the constant 0.1 MPa isobar. Point (1) represents a potential final state of a system undergoing ISP, with a thermodynamic state of −5 °C and 0.1 MPa, corresponding to 5 °C of supercooling at atmospheric pressure. This level of supercooling effectively reduces metabolic activity without introducing any detrimental effects from pressure^25^.

In the isochoric freezing process (IFP), the system follows the liquidus line from point (0) to point (1′), then to point (2′), and ultimately to the triple point (TP). The final state can be any point along this line, such as (1′) or (2′), depending on where the process is halted.

In an isochoric (constant-volume) system, temperature and pressure are interdependent at equilibrium. Along the liquidus line, ice and solution coexist in thermodynamic equilibrium, making it a fundamental reference for understanding the relationship between phase transitions, pressure, and temperature in isochoric preservation.

At thermodynamic state (1′), the temperature is −5 °C and the pressure is 60 MPa, while at state (2′), the temperature is −10 °C and the pressure is 110 MPa. Lowering the temperature from −5 to −10 °C slows metabolism and enhances preservation; however, it also significantly increases the pressure from 60 to 110 MPa.

Such high pressures can have adverse effects on food quality^37^ and may compromise the viability of cryopreserved tissues, such as livers^70^ and hearts^71^. This trade-off between temperature reduction and pressure increase highlights the need for precise control of isochoric conditions to balance improved preservation with the potential risks of elevated pressure.

Pressures of 60 MPa or below are sufficient for microbial elimination over preservation times^18,19,33,47,72,73^. Furthermore, tissues such as livers^70^ and hearts^71^ can tolerate pressures in the range of 40 to 60 MPa, but higher pressures may have detrimental effects on tissue viability.

These limitations have driven the exploration of the isochoric process in this study, which seeks to achieve lower preservation temperatures while avoiding the challenges associated with extreme pressures. By carefully controlling the volume of pure water in the system and the thermodynamic conditions, this approach aims to optimize preservation while maintaining pressures within biologically and industrially acceptable limits.

The isochoric process developed in this study consists of two distinct stages: An initial progression along the liquidus line and a transition along a constant-pressure (isobaric) line. This sequence is depicted in Figs. 1A−C and 3 allows for precise control over both temperature and pressure.

For example, the process may begin with an isochoric freezing phase, moving from point (0) to point (1′), where the system follows the liquidus line as ice forms and pressure increases. This is then followed by an isochoric supercooling phase, transitioning from point (1′) to point (2).

At point (2), the system achieves the same low temperature as point (2′), but at a significantly lower pressure. This approach enables preservation at lower temperatures while mitigating the adverse effects associated with extreme pressures.

A key challenge in designing the isochoric supercooling process, addressed in this paper, is precisely controlling the isochoric freezing process to stop at point (1′) while continuing to lower the temperature to achieve supercooling. This control is essential for optimizing preservation conditions by minimizing pressure while maintaining the benefits of subfreezing storage temperatures.

Designing the isochoric supercooling processes introduced in this study requires two critical datasets: The first one is the liquidus line for the biological matter and the surrounding solution within the impermeable boundary, which determines the equilibrium relationship between temperature, composition, and pressure during freezing, and the second one is the relative volume of water in the surrounding fluid, which ensures that freezing halts once the desired pressure, induced by freezing, is reached.

The thermodynamic analysis used to develop these datasets is detailed in the Thermodynamic Methods section.

To illustrate the calculations and analysis, several examples are presented.

Figure 3A, discussed earlier, depicts the isochoric processes for pure water along the liquidus line, which separates water from ice Ih. Figure 3B illustrates that during the isochoric supercooling process shown in Fig. 3A, leading to point 2, ~74% of the total volume of the isochoric chamber can be utilized for the preservation of biological matter.

This volume fraction plays a crucial role in determining the system’s thermodynamic behavior, including the pressure and temperature conditions as freezing progresses. The data in Fig. 3 suggests that, to achieve supercooling at thermodynamic point 2 in pure water, the volume occupied by the secluded section is 74% of the total chamber volume, while the volume of water surrounding the secluded section is designed to be 26%.

However, it is important to note that the analysis of supercooling in pure water does not directly apply to biological matter, as biological solutions have different thermodynamic properties and phase behavior.

**Fig. 4:**
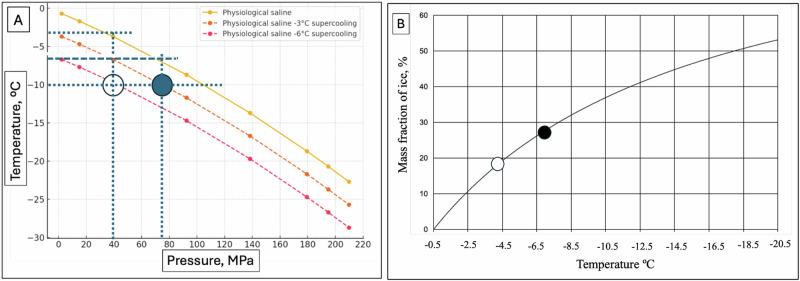
The thermodynamic process of isochoric supercooling in a physiological saline solution. **A** Temperature pressure thermodynamic diagram. It shows the liquidus line between water (top) and ice Ih (bottom). The other two curves show the thermodynamic states at which the water is supercooled by 3 ºC at each pressure and by 6 ºC at each pressure. **B** Similar to 3B and illustrates the process analyzed in this paper.

Figure 4 illustrates the design of an isochoric supercooling process in physiological saline. Figure 4A depicts the liquidus line for physiological saline, derived using equations [8] and [9] from the methods section. Figure 4B shows the percentage of ice mass as a function of the equilibrium temperature along the liquidus line for physiological saline, derived using the mathematical models in the methods section. (Note that this curve is different from that for freezing of a physiological solution in ref. ^10^, because here the composition of the biological matter in the isotonic solution does not change with freezing).

The liquidus line represents the thermodynamic equilibrium state of the isochoric system at the onset of the supercooling step. In any isochoric freezing process, equilibrium is maintained between pure ice and the surrounding solution.

At the start of the supercooling phase, the isochoric system consists of two distinct phases (Fig. 4A): pure ice outside the biological matter and solution boundary, and the matter enclosed within the boundary. Both phases coexist in equilibrium along the liquidus line, ensuring a stable thermodynamic state as the system transitions into the supercooling phase. Supercooling occurs when the temperature is lowered below the liquidus line for a given pressure.

Figure 4A also includes isothermal lines for 3 and 6 °C supercooling. To illustrate the isochoric supercooling process, we will discuss two examples of biological matter preservation at −10 °C:

One scenario with 3 °C of supercooling, and another scenario with 6 °C of supercooling.

These examples will demonstrate how different levels of supercooling influence the thermodynamic conditions of the system and the preservation environment for biological matter.

Figure 4A shows that to preserve physiological saline at −10 °C with 6 °C of supercooling, freezing must stop at −4 °C. At this temperature, the system pressure reaches 40 MPa.

Figure 4B indicates that at −4 °C, the percentage mass of ice is 18%. Therefore, to design a system in which physiological saline is supercooled by 6 °C and preserved at −10 °C, the system must consist of: 82% mass of physiological saline enclosed within an impermeable boundary that allows pressure and heat transfer but prevents mass exchange, and 18% mass of ice, which completely fills the remaining volume of the isochoric chamber.

This configuration ensures that freezing halts at −4 °C, allowing the enclosed physiological saline to remain in a stable supercooled state at −10 °C, while maintaining controlled pressure and thermodynamic stability.

Similarly, Fig. 4A shows that to preserve physiological saline at −10 °C with 3 °C of supercooling, freezing must stop at −7 °C. At this temperature, the system pressure reaches 75 MPa.

Figure 4B indicates that at −7 °C, the percentage mass of ice is 26%. Therefore, to design a system in which physiological saline is supercooled by 3 °C and preserved at −10 °C, the system must consist of: 74% mass of physiological saline enclosed within an impermeable boundary that allows pressure and heat transfer but prevents mass exchange, and 26% mass of ice, which completely fills the remaining volume of the isochoric chamber.

This configuration ensures that freezing halts at −7 °C, allowing the enclosed physiological saline to remain in a stable supercooled state at −10 °C, while maintaining controlled pressure and thermodynamic stability.

**Fig. 5:**
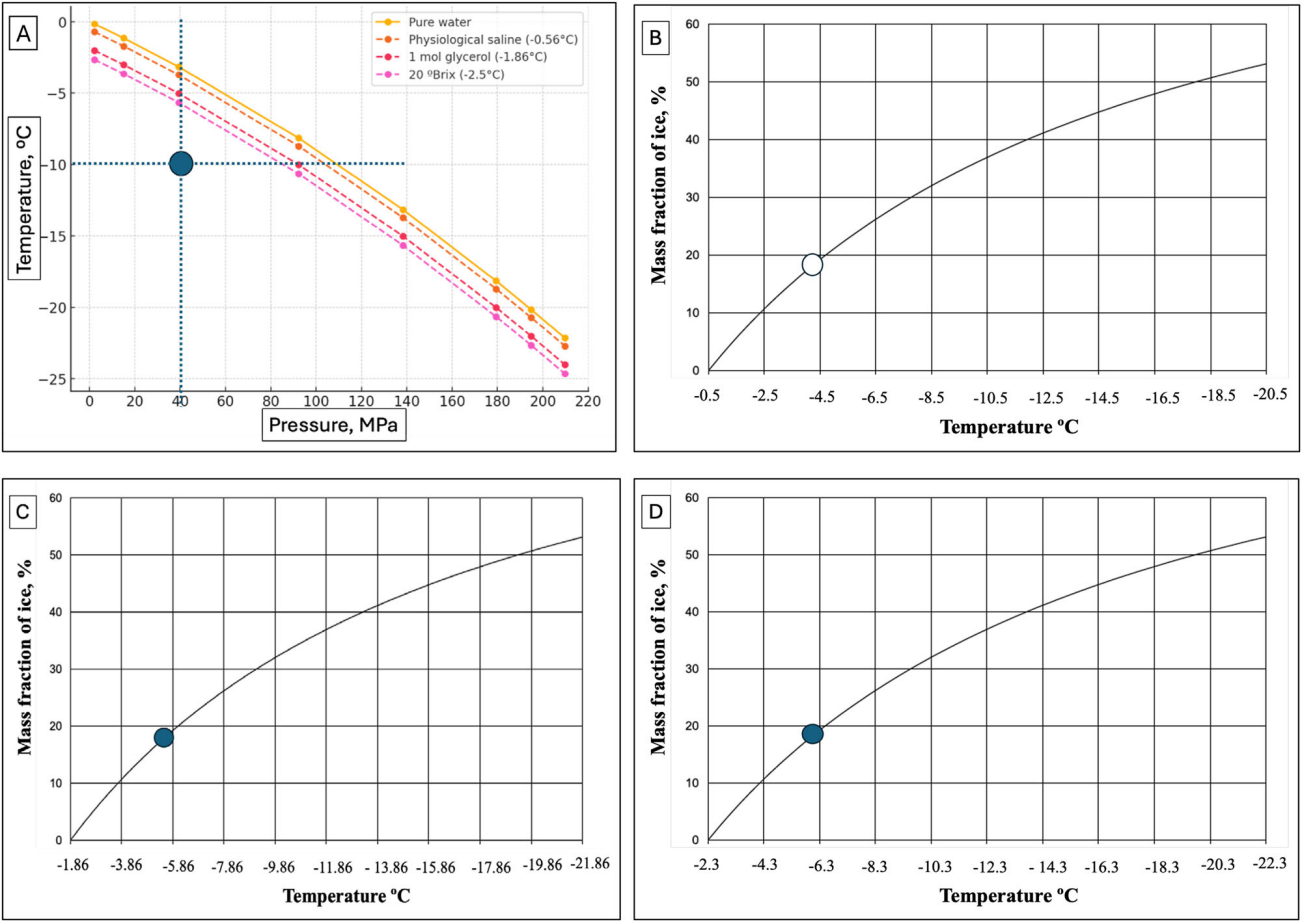
The thermodynamic processes of isochoric supercooling and mass fraction of ice in different solutions. **A** Temperature pressure phase diagram. **B** Mass fraction of ice in physiological saline. **C** Mass fraction of ice in a 1M glycerol solution. **D** Mass fraction in a 20º Brix solution.

Figure 5A, B provides complete information for designing a supercooled isochoric system at the desired supercooling preservation temperature for a biological material in osmotic equilibrium with different solutions. The process follows these steps:

Obtain the phase diagram for the preserved material inside the confined boundary (Fig. 5A).

Plot lines for the desired supercooling based on the phase diagram.

Determine the pressure by drawing a horizontal line from the desired preservation temperature to the liquidus line. The intersection gives the pressure that will be generated at the chosen supercooling and preservation temperature (Fig. 5A).

Determine the percentage of ice by using Fig. 5B, which provides the percentage of ice mass as a function of temperature along the liquidus line for the composition of the preserved material.

By following this approach, one can design an isochoric system that maintains a supercooled state at the target preservation temperature while ensuring precise control over pressure and phase behavior.

Figure 5A presents the liquidus line for pure water, based on data from refs. ^74,75^, along with liquidus lines for physiological saline, 1 M glycerol, and a 20° Brix solution, derived using equations [8] and [9].

The liquidus line represents the thermodynamic equilibrium state of an isochoric system at the onset of the supercooling step. In an isochoric freezing process, equilibrium is maintained between pure ice and the surrounding solution when the system’s thermodynamic parameters lie on the liquidus line.

At the start of the supercooling phase, the isochoric system consists of two distinct phases: pure ice outside the biological matter boundary and the interior within the boundary, both in equilibrium along the liquidus line. This equilibrium condition ensures that the system remains stable as it transitions into the supercooling phase.

The intersection points between the horizontal isothermal −10 °C line and the different liquidus lines indicate the pressures generated solely due to freezing in an isochoric system: Approximately 90 MPa for a 20° Brix solution, approximately 95 MPa for a 1 M glycerol solution, and ~105 MPa for physiological saline solution.

This demonstrates the significant effect of chemical additives in reducing the pressure during typical isochoric freezing processes. By selecting appropriate solutes, it is possible to lower the pressures encountered in isochoric freezing, which may be beneficial for preserving biological matter while minimizing potential pressure-induced damage.

Figure 5A shows that a temperature of −10 °C can be achieved by freezing to a pressure of 40 MPa, in combination with supercooling of about 7 °C for physiological saline, about 5 °C for 1 M glycerol and about 4 °C for 20° Brix. This demonstrates the effects of freezing in the combination of isochoric freezing and supercooling, and changes in composition can be used to facilitate supercooling preservation at a desired temperature with significantly lower pressures compared to isochoric freezing alone. In particular, the addition of chemical species lowers the required degree of supercooling needed to reach the desired preservation temperature, thereby improving the stability of the supercooled system. The levels of supercooling in Fig. 5A have been shown to be stable in isochoric systems^22,25,26,28,63^.

For cryobiology applications, studies indicate that biological systems can tolerate pressures of up to 40 MPa for several days, such as livers^76^ hearts^71,77^. This highlights the potential for using the combination of isochoric freezing and isochoric supercooling with chemical additives to achieve effective preservation at lower pressures and temperatures, making this approach highly relevant for biological preservation in isochoric systems.

Figure 5B–D display the percentage mass of ice as a function of the equilibrium temperature along the liquidus line for physiological saline, 1 M glycerol and 20° Brix. It shows that for a preservation temperature of −10 °C and a pressure of 40 MPa, ice must be 18% of the mass of the system, regardless of the composition. (It should be noted that in the diagram we have neglected the effect of the additives on the compressibility of the unfrozen solution.)

The comparison in Fig. 5B–D illustrates the effects of the composition on the process of supercooling in the isochoric chamber. To reach −10 °C in physiological saline, the supercooling is 6 °C, for 1 M glycerol it is 5 °C, and for 20° Brix it is 4 °C.

This comparison also illustrates how the combination of partial freezing and composition can be used to tailor preservation conditions in terms of preservation temperature, pressure, and degree of supercooling. Additionally, it highlights the role of solute concentration in fine-tuning the isochoric supercooling process, providing a means to control both temperature and pressure for optimized biological preservation.

Crucially, the decoupling of pressure from the isochoric supercooling temperature allows independent control over pressure and degree of supercooling. This capability enables the design of elevated-pressure conditions that reduce microbial contamination in food systems without compromising food quality.

### Practical considerations for the implementation of isochoric supercooling systems

In addition to the fundamental thermodynamic differences between isochoric and conventional supercooling, there are several practical considerations that influence the implementation and performance of isochoric supercooling systems. These considerations are critical to the design and operation of such systems, particularly when deployed in real-world storage environments.

As with all chilled preservation systems, isochoric supercooling systems are typically stored within temperature-controlled chambers. These chambers are generally designed to maintain a uniform spatial temperature. However, in practice, the temperature often fluctuates over time due to the operating characteristics of the control system, including cycling behavior, sensor resolution, and feedback response. These fluctuations can span several degrees Celsius, even in well-regulated systems.

The temperature within the isochoric supercooling chamber responds dynamically to these fluctuations. The response depends on the thermal mass of the chamber, the precision of the temperature controller, and the overall system dynamics. As the ambient temperature fluctuates, the amount of ice present in the isochoric chamber may also vary due to intermittent freezing and melting. These phase changes alter the frozen volume fraction, resulting in corresponding changes in internal pressure. Since the isochoric system operates under conditions where pressure and temperature are interdependent, such pressure fluctuations must be anticipated in system design. Specifically, the temperature setpoint of the storage environment must be selected with regard to the maximum pressure tolerance of the isochoric chamber.

Furthermore, the fluctuation-induced freezing and melting have dual consequences. On the positive side, the latent heat associated with ice formation and melting increases the effective thermal mass of the system. This increase enhances the thermal buffering capacity of the chamber and can improve temperature stability within the isochoric environment^78^. However, on the negative side, more energy is required to re-freeze the ice during downward temperature swings compared to conventional supercooling systems, where no phase change occurs. This requirement can reduce or negate the energy savings typically associated with supercooling-based preservation, as previously reported^78,79^.

In light of these considerations, it is recommended that temperature fluctuations in storage environments utilizing isochoric supercooling be minimized as much as possible. This may involve optimizing control algorithms, increasing insulation, or incorporating phase-change-aware thermal modeling into system design. Additionally, the effects of unavoidable temperature swings should be quantitatively assessed to ensure system reliability and performance under realistic operating conditions.

In summary, this paper introduces the concept of combining an isochoric freezing process with an isochoric supercooling process to enable the reduction of storage temperatures in a supercooled system without altering the degree of supercooling. It provides detailed design guidelines for achieving the thermodynamic state of isochoric supercooling. The design allows for precise control over pressure within the isochoric system, maintaining it below the pressures typically observed in an isochoric freezing process at the same preservation temperature. This capability makes the multiphase isochoric supercooling process a promising approach for optimizing biological preservation while mitigating the challenges associated with high pressures in traditional isochoric freezing.

## Methods

### Thermodynamic analysis

Experiments conducted using a nucleation detection system developed by Consiglio et al.^64^ led to the development of a statistical model to predict the probability of ice nucleation in isochoric systems as a function of the degree of supercooling^26^. Empirical expressions for the probability of ice nucleation (*Pr*) as a function of preservation temperature (*T*) were derived from experimental data for specific systems using Poisson statistics.

Consiglio’s work established a link between the degree of supercooling and the ice nucleation rate (J), which was then related to the freezing probability using Poisson statistics. This analysis demonstrated that the probability of freezing (*Pr*) follows a Poisson distribution. The probability of ice nucleation (*Pr*) as a function of storage temperature (*T*) and time (*t*) is governed by the following relationship:

1 $$Pr \left(T,t\right)=1-{e}^{-{\rm{\gamma }}{({T}_{{eq}}(C,P)-T)}^{n}t}$$

where *Teq* is the equilibrium phase transition temperature, which depends on the system’s composition (*C*) and pressure (*P*). The parameters *γ* and *n* are empirical fitting constants determined experimentally for the specific system. Equation (1) indicates that the supercooling temperature can be lowered without affecting the nucleation probability by decreasing *Teq*(*C*, *P*).

In this equation: *γ* and *n* are nucleation rate parameters determined experimentally, for example, through constant cooling nucleation experiments^26^. *t* represents the duration of supercooling.

Typical values observed in supercooling experiments are *γ* = 10⁻²⁴ K⁻ⁿ⋅s⁻¹ and *n* = 25^67^. However, *γ*and *n* may also depend on pressure, making it essential to consider pressure effects when applying these parameters. Research has shown that increasing pressure can stabilize the supercooled state^80,81^, suggesting that values for *γ* and *n* determined near thermodynamic equilibrium may overestimate nucleation probability under higher pressures. Nevertheless, further studies are needed to accurately assess the effects of pressure, composition, and isochoric chamber design on these parameters to improve the predictive accuracy of nucleation behavior.

The degree (or magnitude) of supercooling in a system is defined as the temperature difference, Δ*T* *=* *Teq* − *T*, where *Teq* is the thermodynamic equilibrium phase transition temperature and *T* is the preservation temperature. The likelihood of nucleation, as expressed in Eq. (1), is approximately a function of Δ*T*. This suggests that thermodynamic states with the same degree of supercooling but different *Teq* can exhibit similar supercooling stability—a measure of the system’s ability to remain in a liquid state below its freezing point.

By lowering the equilibrium freezing temperature (*Teq*) while maintaining the same degree of supercooling (Δ*T* *=* *Teq* − *T*), it becomes possible to achieve preservation at lower temperatures without increasing the probability of nucleation.

**Fig. 6:**
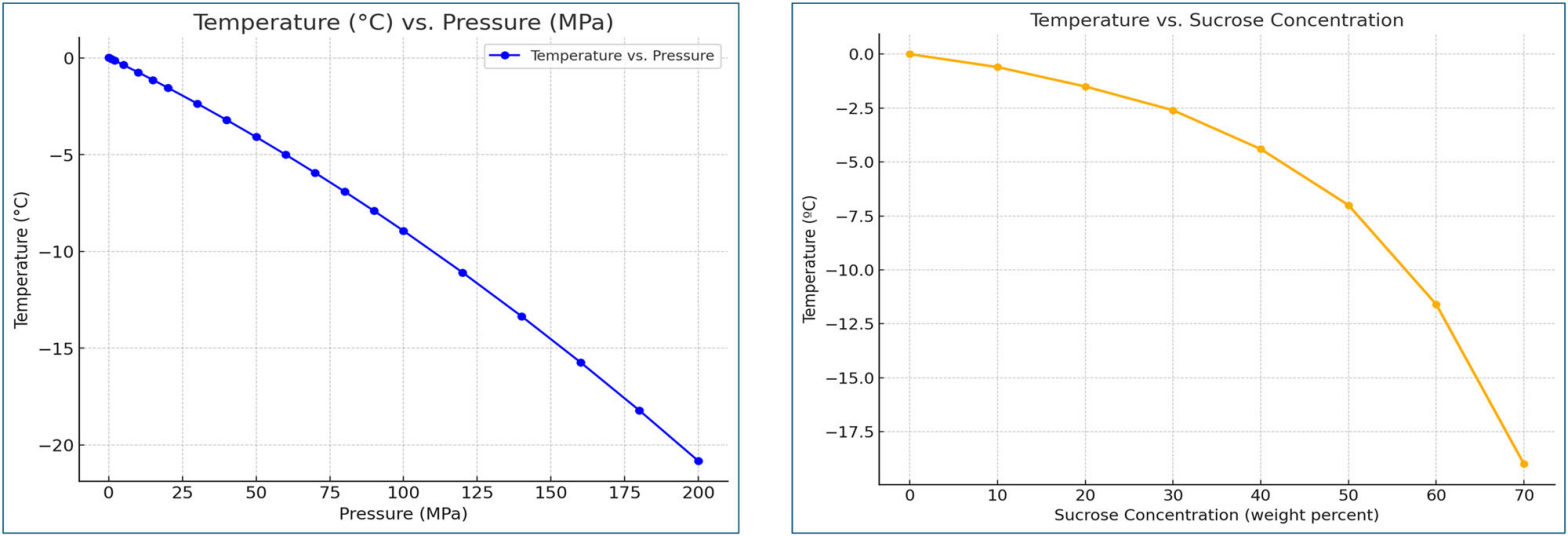
Temperature-pressure liquidus line for pure water^82^ and for sucrose^83^.

Under atmospheric pressure, water freezes at 0 °C. However, two thermodynamic equilibrium-based mechanisms can lower the freezing temperature below 0 °C: The first one is increasing Pressure: Raising the pressure depresses the freezing point within the range from 0 °C to the triple point of water, ice I, and ice III (−21.985 °C, 209.9 MPa), as shown in Fig. 6A (plotted using data from Engineering Toolbox). The second one is altering Composition: Modifying the composition of biological matter can also reduce the freezing temperature. For example, Fig. 6B illustrates freezing point depression caused by the addition of sugar, a common food preservation compound (plotted using data from ref. ^82^).

These mechanisms highlight the potential for controlling freezing behavior in various applications, such as food preservation and cryobiology. In an isochoric freezing system, lowering the thermodynamic equilibrium temperature by increasing pressure and altering composition enables the preservation of biological matter in a supercooled state at lower temperatures without increasing the probability of ice nucleation. The effect of pressure alone on lowering the equilibrium phase transition temperature in an isochoric freezing system with pure water was previously studied by ref. ^67^. Building on that work, this paper investigates the combined effect of increasing pressure and modifying composition on supercooling in isochoric freezing systems.

Designing isochoric freezing and supercooling processes requires two critical datasets: The liquidus line for the biological matter and the surrounding solution within the impermeable boundary, which defines the equilibrium relationship between temperature, composition, and pressure during freezing. And the relative volume of water in the surrounding fluid, which ensures that freezing ceases once the target pressure, induced by freezing, is achieved.

The following section outlines the methodology used to generate these essential datasets, which are fundamental for the precise control and optimization of isochoric processes.

The relative volume of pure water in the isochoric system depicted in Fig. 1B, denoted as *V₁*, required to achieve a specific isochoric-induced pressure, can be determined using the principles of mass conservation in a closed-volume system and the thermodynamic properties of ice and solutions. The mass conservation equation is expressed as follows:

2 $${m}_{t}={m}_{i}+{m}_{b}+{m}_{s}$$

where: *m*_*t*_ is total mass in the isochoric chamber, *m*_*i*_ is mass of ice, *m*_*b*_ is mass of biological matter,

and *m*_*s*_ is the mass of the solution within the enclosed volume.

This equation ensures that the mass contributions from ice, biological matter, and the solution are fully accounted for within the total mass of the isochoric system. By applying this relationship, along with the known thermodynamic properties of the materials and the system’s geometry, the required relative volume of water (*V₁*) can be precisely determined to achieve the desired freezing-induced pressure.

In an isochoric system, the conservation of volume (*V*) applies, which can be expressed as:

3 $${V}_{t}={V}_{i}+{V}_{b}+{V}_{s}$$

Where, *Vt* is the total volume of the isochoric chamber (constant), *V*_*i*_ is the volume of ice formed, V_bs_ is the volume of the biological matter (Vb), and *V*_*s*_ is the volume of the solution enclosed within the impermeable boundary.

This equation ensures that the total system volume remains constant during freezing, as the combined volumes of ice, biological matter, and solution must collectively account for the chamber’s total capacity. This relationship is crucial for calculating the isochoric freezing-induced pressure. Specifically, Equation [3] applies to an isochoric (constant-volume) thermodynamic system, where volume constraints dictate pressure and phase behavior during freezing.

An important thermodynamic variable in this analysis is the quality, *Q*_*m*_*(T)*, which represents the mass fraction of ice in thermodynamic equilibrium at a given temperature (*T*). It is defined as:

4 $${Q}_{m}(T)=\frac{{m}_{i}(T)}{{m}_{t}}$$

where: *Q*_*m*_(*T*) is the mass fraction (quality) of ice at temperature *T*, m_i_ is the mass of ice, and *m*_*t*_ is the total mass in the isochoric system.

The quality, *Q*_*m*_*(T)*, describes the proportion of the system’s mass that exists as ice at a specific temperature. In an isochoric system, as the temperature decreases, *Q*_*m*_*(T)* increases along the liquidus line, corresponding to the pressure rise induced by freezing. This parameter is essential for predicting the system’s thermodynamic behavior and determining the extent of freezing required to achieve the desired pressure in the system.

The quality can also be defined in terms of the volume fraction, *Q*_*v*_*(T)*, which is expressed as the ratio of the volume of ice, *V*_*i*_*(T)*, to the total volume, *V*_*t*_, of the isochoric system:

5 $${Q}_{V}\left(T\right)=\frac{{V}_{i}(T)}{{V}_{t}}$$

where: *Q*_*V*_(*T*) is the volume fraction of ice at temperature *T*, *V*_*i*_(*T*) is the volume of ice formed at temperature *T*, and *V*_*t*_ is the total (constant) volume of the isochoric system.

This volume-based definition of quality provides an alternative perspective on the extent of ice formation within the system, complementing the mass fraction *Q*_*m*_*(T)*. Both definitions are valuable for analyzing and modeling the thermodynamic behavior of isochoric systems, particularly in determining the relationship between temperature, pressure, and the extent of freezing.

The isochoric freezing process occurs along the liquidus line, where temperature and pressure are interdependent. As a result, the thermodynamic state can be defined by either temperature or pressure, along with the system’s quality. The most straightforward method for calculating quality involves the use of specific volume (*υ*), with subscripts *t*, *i*, *b*, and *s* representing the total, ice, biological matter, and solution states, respectively. While specific volume depends on both temperature and pressure, the total specific volume of the isochoric system remains conserved.

The derivation proceeds as follows:

6 $${\upsilon }_{t}=\frac{{V}_{t}}{{m}_{t}}=\frac{{V}_{i}+{V}_{s}+{V}_{b}}{{m}_{t}}$$

There are two approaches to simplifying the calculations for this equation: Neglecting the mass of the surrounding solution. Assuming that the mass of the solution surrounding the biological matter is negligible is a practical and often reasonable approximation. And assuming identical specific volumes for the solution and biological matter: If the specific volumes of the solution and biological matter are approximately the same, they can be treated as a single entity, simplifying the analysis.

Using this assumption, the subscript *bs* is introduced to represent the combined average thermodynamic properties of the biological matter and the surrounding solution. The combined volume is denoted as *V*_*bs*_, and the combined mass is denoted as *m*_*bs*_.

With these terms, Equation [5] simplifies to:

7 $$\begin{array}{l}{\upsilon }_{t}=\frac{{V}_{t}}{{m}_{t}}=\frac{{V}_{i}+{V}_{{bs}}}{{m}_{t}}=\,\frac{{m}_{i}{V}_{i}}{{m}_{t}{m}_{i}}+\frac{\left({m}_{t}-{m}_{i}\right){V}_{{bs}}}{{m}_{0}{m}_{{bs}}}\\\quad={Q}_{m}(P,T){\nu }_{i}(T,\,P)+(1-{Q}_{m}(P,T)){\nu }_{{bs}}(T,P)\end{array}$$

where υ_bs_ represents the combined average specific volume of the biological matter and the surrounding solution.

It should be emphasized that Equation [6] can be solved precisely through an iterative approach. However, this requires additional design considerations, particularly decisions regarding the distribution of mass between the solution and the biological material within the enclosure. Alternatively, using Equation [7] provides a simplified yet effective method that captures the fundamental principles of the design methodology

It is important to note that the specific volumes of the individual components (υ_i_, υ_b_, and υ_s_) are functions of temperature (*T*) and pressure (*P*). However, in a constant-volume, closed control system, the total specific volume (*υₜ*) remains conserved, regardless of the state variables.

Equation (6) can be rearranged to express quality in terms of specific volumes, which are measurable thermodynamic properties. The rearranged form is:

8 $${Q}_{m}(P,\,T)=\frac{{\nu }_{t}-{\nu }_{{bs}}}{{\nu }_{i}-{\nu }_{{bs}}}$$

A few important observations should be noted:

Applicability of the derivation: This derivation is valid only for an isochoric system that satisfies Eq. (2), ensuring conservation of volume within a closed, constant-volume system.

Physical meaning of the term (*υ*_*i*_ *− υ*_*bs*_): This term is positive only when water freezes to ice *Ih*, where density decreases upon solidification, or in scenarios where *υ*_*bs*_ < *υ*_*i*_. In other cases, this term lacks physical significance.

${Q}_{V}(P,T)$ can be derived from Eq. (8)

9 $${Q}_{V}\left(P,T\right)=\frac{{V}_{i}(P,T)}{{V}_{t}}=\frac{{Q}_{m}\left(P,T\right){m}_{t}{\nu }_{t}}{{V}_{t}}=\frac{{\nu }_{i}}{{\nu }_{t}}\left(\frac{{\nu }_{t}-{\nu }_{{bs}}}{{\nu }_{i}-{\nu }_{{bs}}}\right)$$

The thermodynamic state of the biological matter enclosure preserved under isochoric conditions, within the unfrozen portion of the system, is fully defined by the thermodynamic parameters of temperature (or pressure) and quality.

To determine the mass or volume of the unfrozen fraction, it is essential to know the specific volume (or density) of both the ice and the solution along the liquidus line as a function of pressure and temperature. These values enable accurate calculations of the remaining unfrozen fraction based on the conservation of mass and volume, providing a comprehensive description of the system’s thermodynamic state.

Much of the necessary data on the specific volume (density) of various solutions relevant to this process is currently unavailable. However, some data is available from existing studies. For example, the specific volume of water-glycerol mixtures in the temperature range of 15 to 30 °C can be calculated using equations provided by refs. ^83,84^. Additionally, the density of binary solutions and mixtures containing sucrose, glucose, fructose, citric acid, malic acid, pectin, and inorganic salts is documented by ref. ^85^ Furthermore, the effect of pressure on the density of sugar solutions (at 20 °C and up to 300 bar) is detailed in ref. ^86^.

These existing datasets provide valuable starting points, but significant experimental research is still needed to advance the field of biological matter preservation in isochoric systems at subfreezing temperatures. Comprehensive studies on the specific volume and density of relevant solutions under varying temperature and pressure conditions are essential for enhancing the precision and applicability of this preservation method.

Extensive data is available for water, making it the primary medium used to illustrate calculations for the isochoric hypercooling process. Several literature sources^12,13,74,75,87–89^, provide detailed data on the density and specific volume of ice and water along the liquidus line during the freezing of pure water.

Figure 1B, adapted from ref. ^10^, illustrates the fraction of water in an isochoric system along the liquidus line as a function of temperature, extending to the triple point. These figures were derived using correlations for the specific volumes of water as functions of pressure and subfreezing temperature, based on the work of ref. ^90^.

In addition to data on specific volume, another critical thermodynamic dataset required for designing the analyzed process is the temperature-pressure phase diagram of the preserved biological material. Specifically, the liquidus line, where ice Ih and the preserved solution coexist in thermodynamic equilibrium, is essential for accurately modeling and optimizing the isochoric freezing process.

Extensive data on the pure water/ice phase diagram is widely available in the literature, encompassing both experimental studies and quantum simulations (e.g., ref. ^91^). The temperature-pressure relationship for ice *Ih* and pure water in thermodynamic equilibrium, as shown in Fig. 1A, is derived from data provided in refs. ^74,75^.

Phase diagrams for various aqueous solutions coexisting with ice Ih can also be found in studies such as refs. ^88,92^. Additionally, freezing point depression data for various carbohydrates (e.g., sugars) is presented in Fig. 1 of ref. ^93^, providing valuable insights into the behavior of these solutions under subfreezing conditions. These datasets form a critical foundation for analyzing and designing isochoric processes.

In most of our studies, the biological matter is placed in a solution isotonic to the matter^32,37,46,94^. This approach allows preservation conditions to be tailored based on the specific isotonic solution in which the biological matter is immersed, ensuring optimal compatibility and stability during the isochoric process.

When experimental data is unavailable, a commonly used approximation for plotting the liquidus line (i.e., the freezing point depression as a function of pressure and solute concentration) is expressed as:

10 $${T}_{{ph}}\left(P,{C}_{i}\right)={T}_{0}+\Delta T\left(P\right)+\sum \Delta T({C}_{i})$$

Where *T*_*ph*_(*P*, *C*_*i*_) is the phase transition temperature as a function of pressure (*P*) and the concentration of the various species in the solution (*C*_*i*_), *T₀* is the freezing temperature of the solution at the reference pressure of 1 atm, ∆*T*(*P*)represents the phase transition temperature depression due to an increase in pressure, and ∑ ∆*T*(*C*_*i*_) is the summation of the phase transition temperature depression for all solute concentrations (*C*_*i*_). In this approximation, it is assumed that the effects of pressure and solute concentration on the depression of water’s freezing temperature are independent of each other. This assumption is expected to hold at lower pressures and concentrations. However, at higher pressures and concentrations, these effects may not be linearly additive.

For solutions with a known molality, an approximate expression for the freezing point depression as a function of molality, valid at low concentrations, is given by:

11 $$\triangle T\left(K\right)={K}_{f}m$$

Where ${K}_{f}$ is the molal freezing point depression constant, typically, (−1.86 °C/m) and $m$ is the molal concentration of the solute in the solution.

## Data Availability

All data is available upon reasonable request to the corresponding author.
